# Role of p38 mitogen-activated protein kinase in vascular endothelial aging: Interaction with Arginase-II and S6K1 signaling pathway

**DOI:** 10.18632/aging.100722

**Published:** 2015-01-22

**Authors:** Zongsong Wu, Yi Yu, Chang Liu, Yuyan Xiong, Jean-Pierre Montani, Zhihong Yang, Xiu-Fen Ming

**Affiliations:** ^1^ Laboratory of Vascular Biology, Department of Medicine, Division of Physiology, University of Fribourg, CH-1700 Fribourg, Switzerland

**Keywords:** Aging, Arginase-II, endothelial, p38mapk, SASP, S6K1

## Abstract

p38 mitogen-activated protein kinase (p38) regulates cellular senescence and senescence-associated secretory phenotype (SASP), i.e., secretion of cytokines and/or chemokines. Previous work showed that augmented arginase-II (Arg-II) and S6K1 interact with each other to promote endothelial senescence through uncoupling of endothelial nitric oxide synthase (eNOS). Here we demonstrate eNOS-uncoupling, augmented expression/secretion of IL-6 and IL-8, elevation of p38 activation and Arg-II levels in senescent endothelial cells. Silencing Arg-II or p38α in senescent cells recouples eNOS and inhibits IL-6 and IL-8 secretion. Overexpression of Arg-II in young endothelial cells causes eNOS-uncoupling and enhances IL-6 and IL-8 expression/secretion, which is prevented by p38 inhibition or by antioxidant. Moreover, p38 activation and expression of IL-6 and KC (the murine IL-8 homologue) are increased in the heart and/or aortas of wild type (WT) old mice, which is abolished in mice with Arg-II gene deficiency (Arg-II^−/−^). In addition, inhibition of p38 in the old WT mice recouples eNOS function and reduces IL-6 and KC expression in the aortas and heart. Silencing Arg-II or p38α or S6K1 inhibits each other in senescence endothelial cells. Thus, Arg-II, p38, and S6K1 form a positive circuit which regulates endothelial senescence and cardiovascular aging.

## INTRODUCTION

Aging is a major risk factor for cardiovascular disease and is associated with increased oxidative stress and accumulation of senescent endothelial cells in the vasculature [1-3]. Senescent endothelial cells are dysfunctional and reveal uncoupling of endothelial nitric oxide synthase (eNOS) activity, resulting in enhanced superoxide anion production and decreased NO bioavailability [3]. Senescent endothelial cells also exhibit increased inflammatory responses, including enhanced expression of adhesion molecules such as vascular cell adhesion molecule-1 (VCAM-1) and intercellular adhesion molecule-1 (ICAM-1) [4] and adoption of a secretory phenotype that is senescence-associated inflammatory secretion of cytokines and/or chemokines such as IL-6, IL-8, etc, a phenomenon termed senescence-associated secretary phenotypes (SASP) [5]. Studies demonstrate that senescent endothelial cells accumulate in the blood vessels with age [6, 7] and also in atherosclerosis [8, 9], which may importantly contribute to age-accelerated atherogenesis. The mechanisms of cell senescence are multifaceted. Studies show that p38mapk (p38) is an important signaling pathway mediating the senescence process [10-12]. p38 has four mammalian isoforms, α, β, δ, γ, which are expressed to different extent in specific cells and tissues and function as sensors to various stressors, including oxidative stress, inflammatory stimuli, oncogenes, etc. [13]. p38 is activated by dual phosphorylation mediated by the upstream kinases, i.e., MAPK kinases, MKK3 and MKK6 [13] and has been shown to play an essential role in cell senescence and SASP [14, 15]. Of the four enzyme isoforms, p38α is the best recognized isoenzyme of cardiovascular importance [16]. Pharmacological inhibition of p38 reduces atherosclerosis burden and improves plaque stability in animal models [17, 18] and protects against ischemic myocardial injury [16, 19], which can be partly attributed to inhibition of inflammatory responses and foam cell formation [20-23]. In endothelial cells, p38 has been reported to mediate apoptosis and senescence [24, 25]. Inhibition of p38 activity in animals and humans is able to improve endothelial mediated vascular relaxations in diseases [20, 26, 27], suggesting a role of p38 in endothelial dysfunction and senescence.

Our previous study provides evidence that the L-arginine ureahydrolase arginase-II (Arg-II) plays a causative role in uncoupling of eNOS, resulting in oxidative stress, decreased NO bioavailability, enhanced expression of inflammatory adhesion molecules such as VCAM-1 and ICAM-1, and endothelial senescence [4]. This effect of Arg-II in endothelial senescence and age-associated vascular dysfunction involves a positive crosstalk between Arg-II and mTORC1-S6K1 pathway [4]. Excessive activation of mTORC1-S6K1 signaling is implicated in acceleration of aging process and age-related diseases including cardiovascular disease and type 2 diabetes mellitus in various animal models [28] and has been demonstrated to be responsible for loss of replicative/regenerative potential and hypertrophy during cell senes-cence, a process called “geroconversion” [29-32]. Vascular Arg-II expression and activity are increased in aging and obesity mouse models [4, 27]. Although Arg-II, p38, or mTORC1-S6K1 and a crosstalk between Arg-II and mTORC1-S6K1 or between Arg-II and p38 are reported to play a role in endothelial dysfunction in aging and obesity [4, 27], an interaction among the three pathways in vascular endothelial aging and endothelial SASP function is not known. This aspect is therefore investigated in our current study.

## RESULTS

### Enhanced Arg-II and p38 signaling and cytokine secretion in senescent ECs

**Figure 1:**
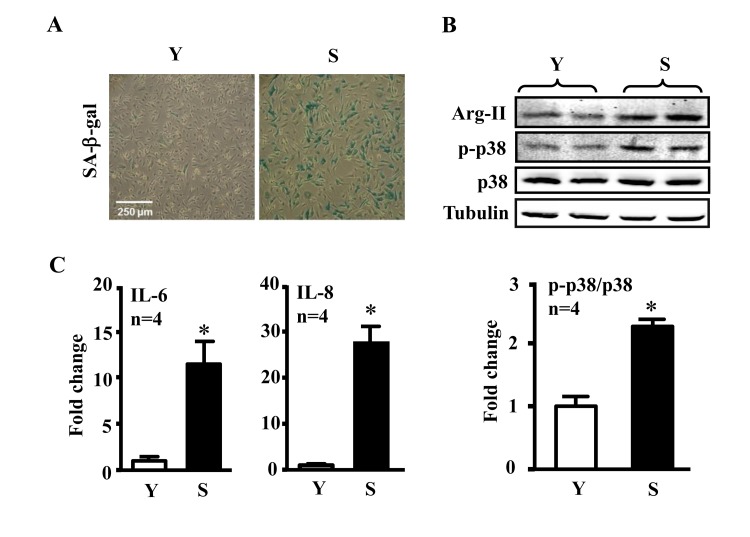
Comparison of inflammatory cytokines between young and senescent endothelial cells Conditioned medium and cell lysate were collected from young (Y) and senescent (S) HUVECs which were serum-starved overnight. **(A)** SA-β-gal staining. **(B)** Immunoblotting to detect Arg-II, phsopho-p38-Thr180/Tyr182 (p-p38) and total-p38 (p38) in young and senescent cells. Tubulin served as loading control. **(C)** The secretion of IL-6 and IL-8 was evaluated by ELISA with collected conditioned medium described above. n=4, *p<0.05 vs young cells (Y).

Our previous study showed increased Arg-II expression and activity in the senescent ECs [4]. We confirmed this result in our current study and showed further that the senescent ECs which reveal increased activity of SA-β-gal (Fig. 1A), had also enhanced activation of p38 as demonstrated by increased levels of phosphorylated p38, i.e., p38-Thr180/Tyr182 (p-p38) as compared to the young cells (Fig. 1B). The increased activation of Arg-II and p38 was associated with enhanced secretion of the inflammatory cytokines IL-6 and IL-8 measured in the conditioned medium from the senescent cells as compared to that from the young cells (Fig. 1C).

### Arg-II-p38 crosstalk promotes eNOS-uncoupling and cytokine production in senescent ECs

**Figure 2:**
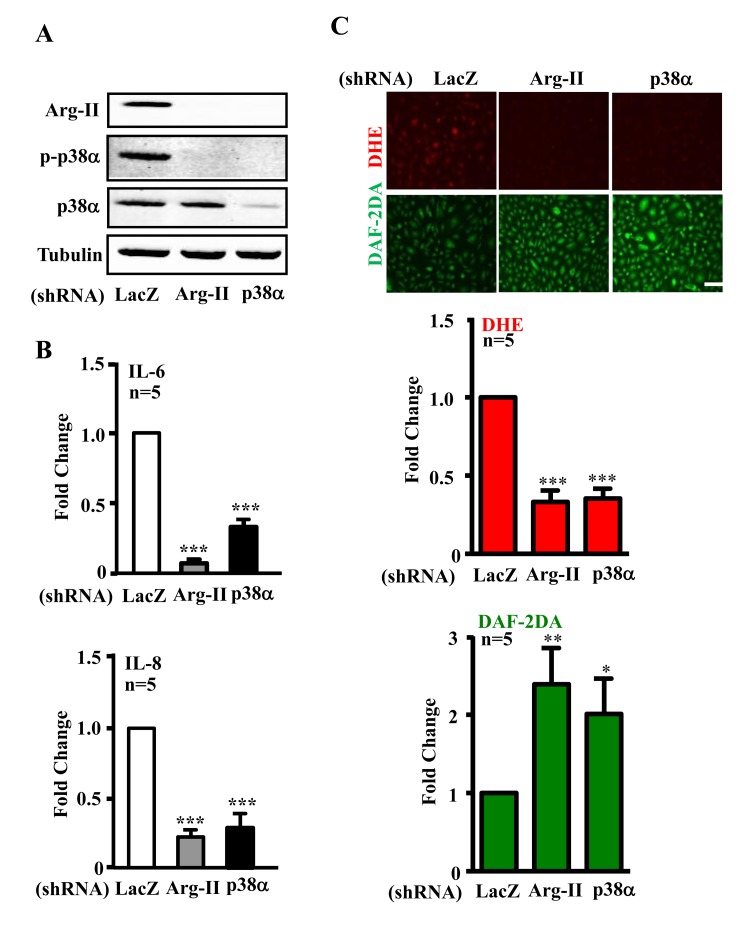
Silencing Arg-II or p38α reduces cytokine/chemokine secretion and recouples eNOS in senescent endothelial cells Senescent HUVECs were transduced with control shRNA (rAd/U6-LacZ^shRNA^), rAd/U6-Arg-II^shRNA^ or -p38α^shRNA^. Ninety six hours post transduction, cells were serum-starved overnight. Cell culture conditioned medium were collected for ELISA, and cell extracts were subjected to immunoblotting. **(A)** Immunoblotting analysis of Arg-II, phsopho-p38α–Thr180/Tyr182 (p-p38α) and total-p38α(p38α). Tubulin served as loading control. **(B)** Cytokine/chemokine secretion was determined by ELISA with collected conditioned medium. **(C)** DHE and DAF-2DA staining for detection of superoxide anions and NO, respectively. Quantification of the signals were presented in the bar graphics. n=4, *p<0.05, **p<0.01, ***p<0.001 vs shRNA-LacZ group. Scale = 200 μm.

We further investigated whether Arg-II and p38 interact with each other and promote cytokine secretion in senescent endothelial cells. As shown in Fig. 2A, shRNA-mediated silencing of Arg-II in the senescent endothelial cells inhibited activation of p38α, the proapoptotic isoform of p38 in human endothelial cells [33]. Conversely, silencing p38α in the cells also inhibited Arg-II expression. The results implicate a positive crosstalk between Arg-II and p38 signaling. The production of IL-6 and IL-8 in senescent ECs as measured in the conditioned medium was reduced by silencing either Arg-II or p38α (Fig. 2B). Moreover, silencing either Arg-II or p38α in the senescent endothelial cells decreased superoxide anion production (DHE staining) and increased NO signal (DAF-2DA staining) (Fig. 2C), demonstrating a role of Arg-II-p38 crosstalk in endothelial dysfunction.

**Figure 3:**
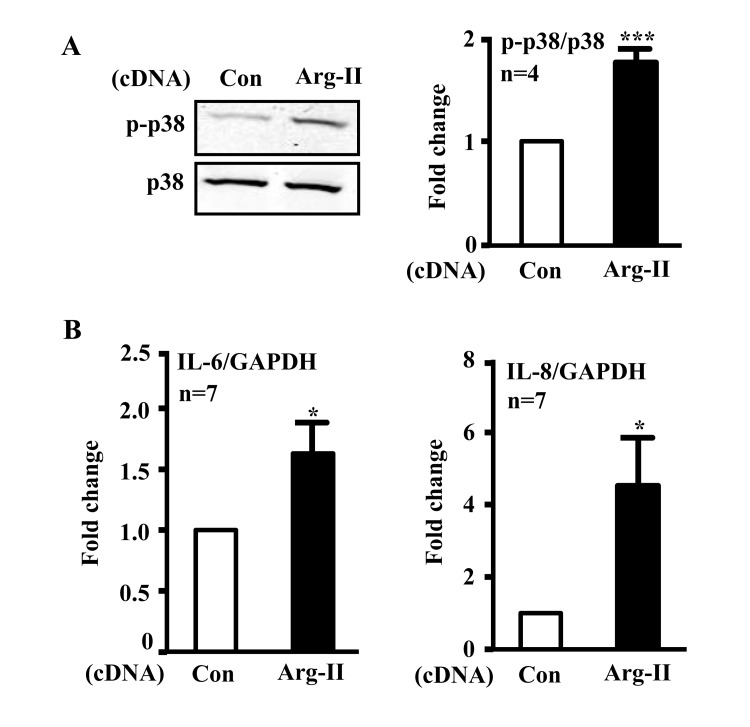
Overexpression of Arg-II in young endothelial cells activates p38 and enhances secretion of IL-6 and IL-8 HUVECs were transduced with rAd empty vector as a control or rAd/CMV-Arg-II. Forty eight hours post transduction, cells were serum-starved overnight. Cell culture conditioned medium was collected for ELISA and cell extracts were subjected to immunoblotting. **(A)** Immunoblotting analysis of phsopho-p38-Thr180/Tyr182 (p38) and total-p38 (p38). **(B)** Cytokine secretion was determined by ELISA with collected conditioned medium. n=4, *p<0.05, ***p<0.001 vs control group (Con).

**Figure 4:**
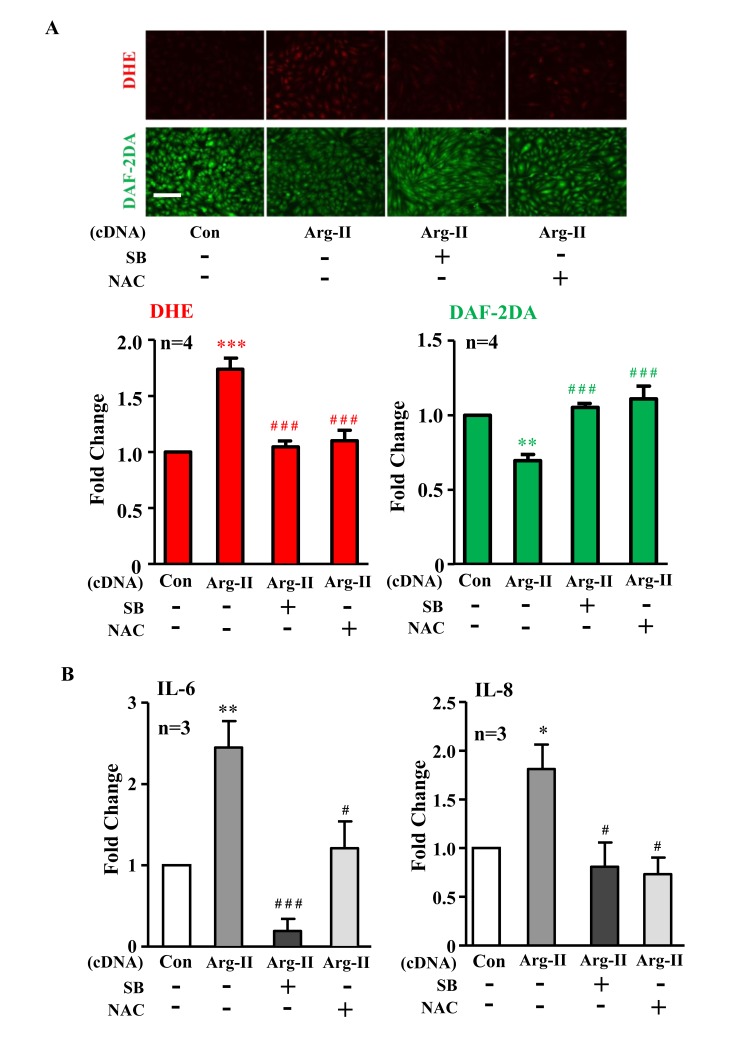
Inhibition of p38 or reactive oxygen species (ROS) prevents Arg-II-induced eNOS-uncoupling and SASP Young HUVECs were transduced with rAd empty vector as a control (Con) or rAd/CMV-Arg-II. The p38 inhibitor SB203580 (10 μmol/L) and ROS scavenger N-acetyl-cystein (NAC, 5 mmol/L) were added right after transduction. **(A)** DHE and DAF-2DA staining for detection of superoxide anion and nitric oxide (NO), respectively. Quantification of the signals is presented below, n=4. **(B)** Cytokine production detected by ELISA in conditioned medium. n=3. *p<0.05, **p<0.01, ***p<0.001 vs control group (Con); #p<0.05, ###p<0.001 vs Arg-II group. Scale = 200μm.

In addition, ectopic expression of Arg-II cDNA in young cells increased p38-Thr180/Tyr182 levels (Fig. 3A) and enhanced IL-6 and IL-8 expression (Fig. 3B). Treatment of the cells with the p38 inhibitor SB203580 (10 μmol/L) or with the anti-oxidant N-acetyl-cysteine (NAC) restored eNOS function (i.e., inhibited superoxide generation and enhanced NO production) (Fig. 4A) and inhibited IL-6 and IL-8 secretion induced by Arg-II overexpression in the young endothelial cells (Fig. 4B).

### Arg-II deficiency prevents p38 activation and vascular cytokine production in aged mice

**Figure 5:**
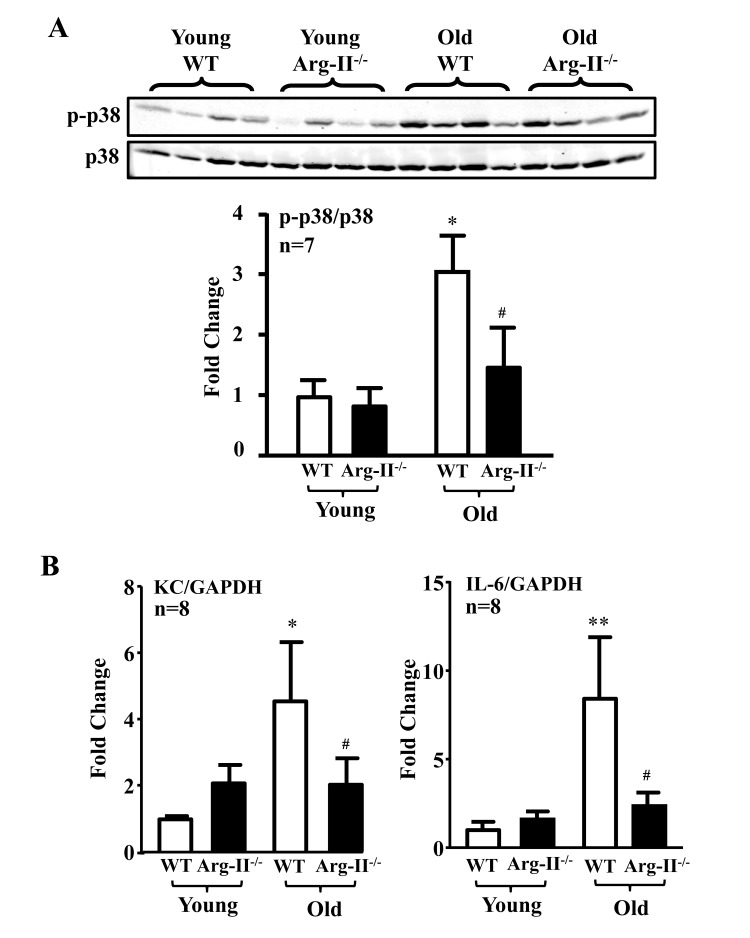
Arg-II gene deficiency in mice (Arg-II−/−) prevents age-associated enhanced p38 activation and cytokine expression in aortas and/or hearts Aortas and/or hearts isolated from young (3-4 months) and old (23-24 months) WT and Arg-II^−/−^ mice were subjected to **(A)** immunoblotting analysis of phsopho-p38-Thr180/Tyr182 (p-p38) and total-p38 (p38) in aortas. n=7 mice in each group. **(B)** qRT-PCR analysis of KC (the murine IL-8 homolog) and IL-6 expression in the heart of young and old mice. n=8 mice in each group. *p<0.05, **p<0.01 vs young wild type (WT) mice; #p<0.05 vs old wild type (WT) mice.

**Figure 6:**
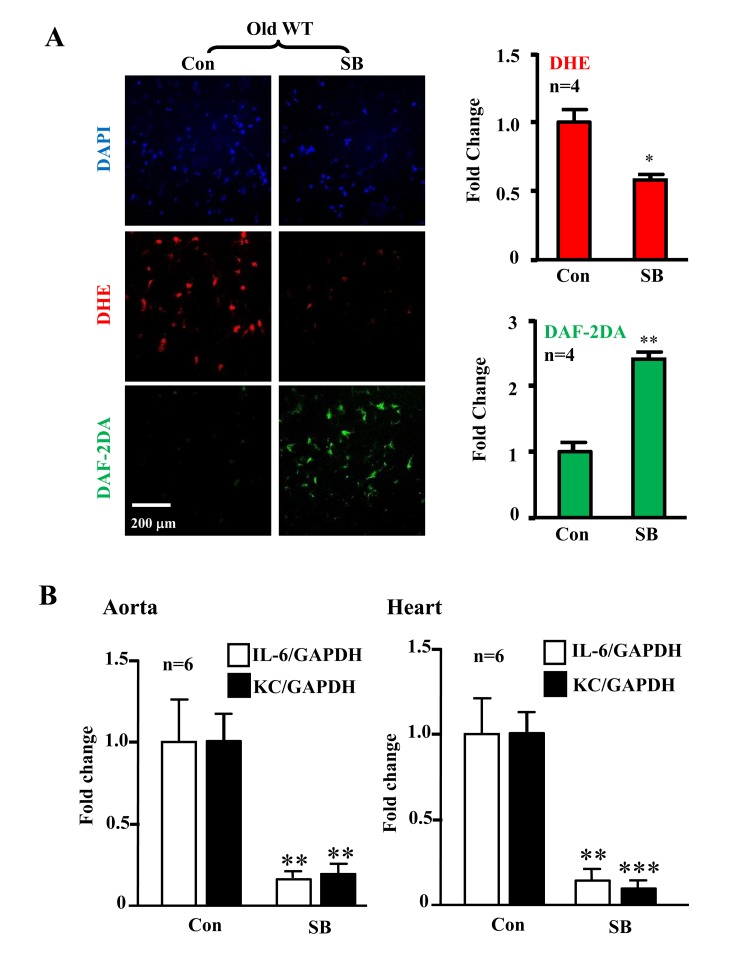
Inhibition of p38 recouples eNOS and reduces cytokine/chemokine expression in aortas/hearts of aged WT mice Treatment of the intact aortas and hearts of old WT mice with SB203580 (10μmol/L, 6 hours) recoupled eNOS function and reduced IL-6 and KC expression. **(A)** *En face* DHE (for detection of superoxide anion) and DAF-2DA (for detection of NO) followed by counter staining with DAPI of the aortas. n=4. **(B)** qRT-PCR analysis of KC and IL-6 in aortas and hearts as indicated. n=6. *p<0.05, **p<0.01, ***p<0.001 vs. control group (Con). Scale = 200 μm.

The levels of p38-Thr180/Tyr182 phosphorylation (pp38) in the aortas were significantly higher in the old WT mice as compared to the aged-matched Arg-II^−/−^ mice, whereas Arg-II deficiency did not affect p38 activation in young mice (Fig. 5A). Gene expression of KC (IL-8 homologue in mouse) and IL-6 in the heart (aortas are not analyzed due to shortage of materials) was significantly elevated in the old mice, which was alleviated in the age-matched Arg-II^−/−^ mice (Fig. 5B). Moreover, treatment of the intact aortas and hearts of old WT mice with SB203580 (10 μmol/L, 6 hours) improved eNOS function, i.e., inhibited superoxide anion production and enhanced NO generation (Fig. 6A), and reduced IL-6 and KC expression (Fig. 6B).

### Crosstalk between Arg-II, p38, and S6K1 pathways in senescence endothelial cells

**Figure 7:**
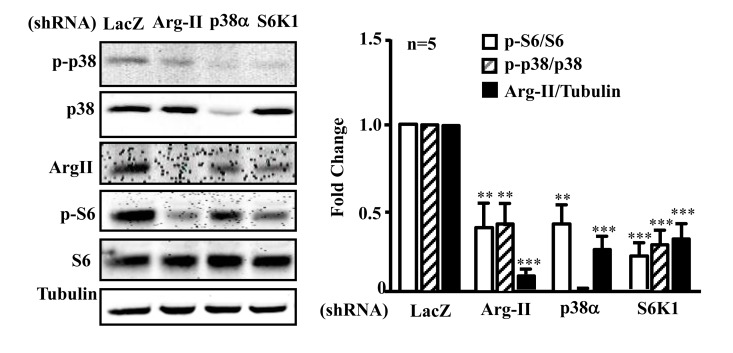
Arg-II, p38, and S6K1 form a positive regulatory circuit in senescent endothelial cells Senescent HUVECs were transduced with rAd/U6-LacZ^shRNA^ as control, -Arg-II^shRNA^, -p38α^shRNA^ or -S6K1^shRNA^. Ninety six hours post transduction, cells were serum-starved overnight. Cell lysates were prepared and subjected to immunoblotting analysis with antibodies against phospho-p38-T180/Y182 (p-p38), total p38 (p38), Arg-II, phosphor-S6-S235/236 (p-S6), and total S6 (S6). Tubulin served as loading control. The bar graph presents the quantification of the immunoblotting analysis. n=5, **p<0.01, ***p<0.001 vs corresponding shRNA-LacZ group.

In senescent endothelial cells in which there is a high activity of p38, Arg-II, and S6K1, silencing Arg-II reduced p-p38 and p-S6 levels (Fig. 7A and 7B).

Silencing S6K1 not only reduced Arg-II expression, but also inhibited activation of p38 (reduced ratio of p-p38/p38). Moreover, silencing p38α also reduced Arg-II expression and S6K1 activity in the senescent endothelial cells (Fig. 7A and 7B). The results demonstrate a positive crosstalk between Arg-II, p38, and S6K1 in senescent endothelial cells.

## DISCUSSION

Aging-associated endothelial dysfunction, particularly, functional defect of eNOS such as eNOS-uncoupling rather than decreased eNOS gene expression is considered as one of the most important mechanisms linking to age-associated cardiovascular diseases [3, 34]. eNOS-uncoupling not only associates with advanced aging or cellular senescence, but also plays a causative role in promoting vascular aging and endothelial cell senescence [4, 35, 36], which is involved in acceleration of vascular diseases including atherosclerosis and diabetic vascular complications [37, 38]. Cell senescence is viewed as a stress response to diverse stimuli, which is manifested by active release of inflammatory cytokines such as IL-6 and IL-8, etc., i.e., SASP, leading to functional alterations of the cells through autocrine or paracrine mechanisms [39]. The novel finding of the present study is the demonstration of a positive crosstalk among Arg-II, p38, and S6K1 signaling pathways, leading to endothelial aging phenotypes including SASP in cultured cells and also in an aging mouse model. Disruption of this positive interplay by inhibiting one of these molecules would provide a novel strategy for treatment of aging-associated cardiovascular diseases.

In line with previous studies [4], our present study first confirmed the important role of Arg-II in aging-associated eNOS-uncoupling in cultured senescent cells and in an aging mouse model. We further showed that increased Arg-II is also responsible for enhanced IL-6 and IL-8 expression and secretion in endothelial aging, since silencing Arg-II in senescent endothelial cells or ablation of Arg-II gene in aged mice not only recouples eNOS function, but also inhibits the cytokine and chemokine expression or secretion. Our previous studies demonstrated that Arg-II promotes endothelial aging through eNOS-uncoupling mechanism which is dependent on the L-arginine-metabolizing effect, since inactive Arg-II mutant does not cause eNOS-uncoupling and endothelial senescence [4]. eNOS-uncoupling is critical for enhanced adhesion molecule expression in aortas of aged mice [4]. The fact that the antioxidant NAC not only recouples eNOS function but also decreases production of IL-6 and IL-8 in cells overexpressing Arg-II, demonstrates that eNOS-uncoupling evoked by Arg-II also plays a crucial role in endothelial SASP.

Emerging evidence demonstrates that p38 is involved in endothelial dysfunction and senescence [26, 40]. Studies including our own demonstrate that p38 is activated in the aortas of angiotensin-II-induced hypertension and in high fat diet-induced obesity [27, 40]. In the present study, we also show significantly augmented p38 activation in senescent endothelial cells and in aged mouse aortas. The augmented p38 activation is involved in Arg-II-induced eNOS-uncoupling and cytokine production in aging, since Arg-II deficiency prevents augmented p38 activation in old mouse aortas and inhibition or silencing of p38α, the isoform mediating endothelial pro-apoptotic responses [33], in senescent endothelial cells and in the aortas of aged mice restores eNOS function and inhibits IL-6 and IL-8 (in humans) and KC (IL-8 homologue in mouse) expression/secretion. Conversely, over-expression of Arg-II in young endothelial cells activates p38, causes eNOS-uncoupling and enhances IL-6 and IL-8 expression, which can be prevented by inhibition of p38. In line with our observation, p38 has been shown to up-regulate cytokines such as IL-6, IL-8, and TNF-α in some biological contexts [41-43]. This result is in agreement with our most recently published study in obesity model showing that p38 is the downstream signal mediating the detrimental effects exerted by Arg-II on vascular endothelial cells [27].

Of note, the relationship of p38 and Arg-II has been reported in the literature. It has been shown that inhibition of p38 reduces arginase activity and Arg-II expression, improves corpus cavernosum tissue relaxation in the angiotensin-II-induced hypertension mouse model [44], suggesting that p38 is the upstream signal that enhances Arg-II expression. These seemly contradictory results can be explained by the results in this study demonstrating a mutual positive crosstalk between Arg-II and p38 in the endothelial cells. This conclusion is supported by the results showing that both Arg-II and p38 are activated in the senescent endothelial cells and inhibition or silencing of the either enzyme reduces the other and is able to restore eNOS function and inhibit IL-6 and IL-8 production from the senescent cells. The question remains how Arg-II and p38 activates each other.

Our previous study demonstrated a crosstalk between Arg-II and S6K1 pathway which plays a causative role in eNOS-uncoupling and acceleration of endothelial senescence and age-associated vascular dysfunction [4]. The relationship between Arg-II, p38, and S6K1 signaling is therefore further postulated and analyzed in the current study. Indeed, in senescent endothelial cells, silencing either of the enzymes could inhibit the other two, which demonstrates a positive regulatory circuit among the three pathways. A crosstalk between p38 and mTOR pathways has been reported [45, 46]. p38α has been shown to activate mTORC1 signaling through its downstream kinase MK2 which phosphorylates TSC2 at serine 1210 residue and prevents TSC2 from inhibiting mTORC1, leading to activation of mTORC1-S6K1 [46]. The effect of p38β seems controversial. It has been reported that p38β either positively or negatively regulates mTORC1 activation through phosphorylation of Raptor on Ser863 and Ser771 [47], or through MK5 (PRAK) which causes phosphorylation of Rheb on Ser130 preventing mTORC1 activation [48], respectively. Our current study provides evidence that it is p38α which interacts with Arg-II and mTORC1/S6K1 pathway, since the p38 inhibitor SB203580 which inhibits p38α and p38β (not p38δ and p38γ) [49] and silencing p38α achieved the same effects on eNOS recoupling, inhibition of SASP and inhibition of Arg-II and S6K1. Conversely, mTORC1 seems also able to activate p38. It has been shown that the mTOR inhibitor rapamycin enhances expression of MKP1, which negatively regulates p38 and leads to reduced p38 activation [50]. It remains to be investigated whether these mechanisms are involved in the crosstalk among Arg-II, p38, and mTORC1 signaling in endothelial aging. It is worth noting that Arg-I exerts similar effects as Arg-II on endothelial dysfunction associated with diabetes and aging animal models [51, 52]. Although there is discrepancy of arginase isoform expression pattern in the vasculature, species difference of the isoform expression exists [53]. Previous studies including our own demonstrated that Arg-II is the dominant isoform expressed in human and mouse endothelial cells, and Arg-I is hardly detectable and is not upregulated in aging [4, 54-56]. It would be interesting to investigate whether Arg-I, similar to Arg-II, induces endothelial dysfunction in species which predominantly express Arg-I such as rats [51, 52] through the same functional cross-talk with p38 and mTORC1/S6K1 in aging.

It is well known that protein kinase B or Akt is the upstream kinase which activates eNOS through phosphorylation of eNOS at S1177 [57-59]. However, chronic activation of Akt has been shown to accelerate endothelial senescence [60], which could be explained by the fact that Akt functions as an upstream signaling for activation of mTORC1-S6K1 [61-63] and sustained elevated Akt activity leads to hyperactive mTORC1-S6K1 signaling which causes eNOS-uncoupling, resulting in endothelial aging [4]. The hyperactive Akt must contribute to the activation of the positive feed-back circuit between Arg-II, p38, and S6K1, leading to eNOS-uncoupling in aging. Hence, disruption of the positive feedback circuit among Arg-II, p38, and S6K1 may have important therapeutic impact on aging-associated cardiovascular diseases.

In conclusion, our study demonstrates a positive crosstalk of the three pathways, i.e., Arg-II, p38mapk, and S6K1 in promotion of eNOS-uncoupling, endothelial aging and inflammation. This mechanism may explain the current ongoing clinical studies which show beneficial effects of p38 inhibitors in treatment of cardiovascular diseases [20, 21, 64].

## MATERIALS AND METHODS

### Materials

RPMI-1640 was purchased from Amimed (Muttenz, Switzerland) ; Endothelial cell growth supplement (ECGS) pack was from PromoCell GmbH (Allschwil, Switzerland) and all cell culture media and materials were purchased from Gibco BRL (Lucerne, Switzerland); BLOCK-iT^TM^ Adenoviral RNAi Expression system was from Life Technologies (Zug, Switzerland); ELISA MAX^TM^ Deluxe sets for human and mouse Interleukin-6 (IL-6), human Interleukin-8 (IL-8), mouse keratinocyte-derived chemokine (KC, the *murine IL-8* homologue) were purchased from Biolegend (Lucerne, Switzerland); p38 inhibitor SB203580 (4-[4-Fluorophenyl]-2-[4-methylsulfinyl-phenyl]-5-[4-pyridyl]1H-imidazole) was purchased from Calbiochem (Lucerne, Switzerland); Bio-Rad DC^TM^ protein assay kit was from Bio-Rad Laboratories (Basal, Switzerland); rabbit anti-phospho-p38mapk (pThr-180/pTyr-182) antibody was from Cell Signaling Technology (Allschwil, Switzerland); and mouse anti-p38mapk antibody was from BD biosciences (Allschwil Switzerland); anti-Arg-II antibody was from Santa Cruz Biotechnol (Basel, Switzerland); N-Acetyl-L-cysteine (NAC) and anti-α-tubulin antibody was from Sigma (Buchs, Switzerland); Alexa Fluor680-conjugated anti-mouse IgG (A21057), dihydroethidium (DHE) were from Molecular Probes/Invitrogen (Lucerne, Switzer-land), and IRDye800-conjugated anti-rabbit IgG (926–32211) were from LI-COR Biosciences (Bad Homburg, Germany); the membrane-permeable 4,5-diamino-fluoresceine diacetate (DAF-2DA) was from VWR international SA (Dietikon, Switzerland).

### Animals

The Arginase II-deficient mice (Arg-II^−/−^) were kindly provided by Dr. William O'Brien [65] and backcrossed to C57BL/6J more than eight generations. Genotyping was performed by polymerase chain reaction (PCR) as previously described [65]. The WT and Arg-II^−/−^ offsprings from hetero/hetero cross were interbred to obtain WT and Arg-II^−/−^ mice, respectively, for experiments. Both WT and Arg-II^−/−^ mice were maintained on a 12-h light-dark cycle and fed standard chow diet and tap water according to the local guidelines of animal experimentation. The female young (2 – 3 months old) and old (20 – 24 months old) animals were anesthetized with xylazin (10 mg/kg body weight, intraperitoneally) and ketamin (100 mg/kg body weight, intraperitoneally) and sacrificed. Heart and thoracic aortas dissected and cleaned from perivascular fat were either subjected to *en face* staining or snap frozen directly in liquid nitrogen and kept at −80°C till further biochemical analyses. To examine the effect of p38mapk, the isolated hearts or/and aortas were treated with vehicle DMSO or SB203580 (10 μmol/L, 6 h) and then snap frozen or subjected to *en face* staining.

### Recombinant adenoviral (rAd) generation

Generation of rAd expressing short hairpin RNA (shRNA) targeting human p38mapk-alpha (p38α) driven by the U6 promoter (rAd/U6-hp38α^shRNA^) was carried out with the Gateway technology (Invitrogen Life Technologies) according to the manufacturer's instructions. rAd/U6-LacZ^shRNA^ was used as controls for rAd/U6-hp38α^shRNA^. The human p38α targeting sequence for rAd/U6-hp38α^shRNA^ is indicated in bold, sense: 5′-CACC**GTTACGTGTGGCAGTGAAGAA**CGAATTCTTCACTGCCACACGTAAC-3′ [3]. The rAd/U6-LacZ^shRNA^, rAd/U6-hArg-II^shRNA^, the empty rAd/CMV and rAd/CMV-mArg-II were described previously [4, 66, 67].

### Human umbilical vein endothelial cell (HUVEC) culture and adenoviral transduction

Preparation and culture of young and senescent HUVEC as well as transduction of HUVEC by recombinant adenovirus were performed as previously describe [3]. Cells at passage 1-3 were deemed as ‘young’ while passage 9-11 as ‘senescent’ cells.

### Senescence-associated β-galactosidase (SA-β-gal) staining

SA-β-galactosidase staining was performed 5 days post transduction as described [4]. Briefly, cells were initially washed twice with PBS followed by fixation with 2% formaldehyde solution in PBS for 10–15 min. After washing twice with PBS, cells were then incubated with the SA-ß-gal staining solution (1 mg/ml X-gal, 40 mmol/L citric acid, 5 mmol/L potassium ferrocyanide, 5 mmol/L potassium ferricyanide, 150 mmol/L sodium chloride, 2 mmol/L magnesium chloride dissolved in phosphate buffer, pH 6.0) overnight at 37^o^C in a CO_2_-free atmosphere. The picture of stained senescent cells was taken under conventional microscopy.

### ELISA

Conditioned medium was collected after serum starvation for overnight. The amount of IL-6 and IL-8 in the conditioned medium was determined with ELISA MAX^TM^ Deluxe kits (Biolegend, San Diego, CA) according to manufacturer's instruction.

### Western blot

Cell lysate preparation, SDS-PAGE, and immunoblotting, antibody incubation and signal detection were performed as described previously [67]. Quantification of the signals was performed using NIH Image 1.62 software.

### Quantitative reverse transcription PCR (qRT-PCR) analysis

IL-6 and IL-8 mRNA expression was determined by two-step qRT-PCR as described previously [67]. Briefly, total RNA was extracted from cells or tissues with Trizol Reagent (Molecular Research Center, Inc., Cincinnati, OH, USA) following the supplier's protocol. First-strand cDNA was synthesized from 500 ng total RNA with a random primer. Real-time PCR was performed with the iQ^TM^ SYBR Green Supermix and iCycler system (Bio-Rad). mRNA expressions were normalized to the reference gene glyceraldehyde 3-phosphate dehydrogenase (GAPDH). PCR primers are listed below:

human IL-6F: GGCACTGGCAGAAAACAACC

human IL-6R: GCAAGTCTCCTCATTGAATCC

human IL-8F: CTGGCCGTGGCTCTCTTG

human IL-8R: CCTTGGCAAAACTGCACCTT

human GAPDH-F: TGCACCACCAACTGCTTAGC

human GAPDH-R: GGCATGGACTGTGGTCATGAG

mouse IL-6F: GACAACCACGGCCTTCCCTA

mouse IL-6R: GCCTCCGACTTGTGAAGTGGT

mouse KC-F : CAATGAGCTGCGCTGTCAGTG

mouse KC-R : CTTGGGGACACCTTTTAGCATC

mouse GAPDH-F : ACCCAGAAGACTGTGGATGG

mouse GAPDH-R: ACACATTGGGGGTAGGAACA

### Detection of NO and superoxide level in cultured endothelial cells

Detection of NO and superoxide levels in cultured endothelial cells was performed as described previously [3]. Briefly, for detection of NO, HUVECs were gently washed twice with Ca^2+^-free PBS, and incubated in a modified Krebs-Ringer bicarbonate solution containing 118 mmol/L NaCl, 4.7 mmol/L KCl, 2.5 mmol/L CaCl2, 1.2 mmol/L MgSO4, 1.2 mmol/L KH2PO4, 25 mmol/L NaHCO3, 0.026 mmol/L EDTA, and 5.5 mmol/L glucose mixed with 5 mmol/L of DAF-2DA for 30 minutes. For measurement of cytoplasmic superoxide, cells were incubated with 5 mmol/L DHE dissolved in culture medium for 30 minutes. The cells were then washed 3 times and images were obtained with Zeiss fluorescence microscopy. The intensity of the fluorescence was quantified by NIH Image J software.

### *En face* Detection of NO and superoxide in rat aortas

NO and superoxide production in the absence or presence of SB203580 was assessed with DAF-2DA and DHE staining, respectively, as described previously [3]. Briefly, old female mice (20-24 months) aortas cleaned of perivascular tissues were equilibrated for 30 minutes in Krebs buffer at 37°C aerated with 95% O_2_ and 5% CO_2_. After equilibration, DMSO or SB203580 (10 μmol/L) were added to respective blood vessels for one hour. DAF-2DA (5 μmol/L) or DHE (5 μmol/L) was then added for 30 minutes or 10 minutes, respectively. The aortas were then washed three times and fixed in 4% paraformaldehyde followed by counter-staining with DAPI (300 nmol/L for 3 minutes). After washing with phosphate buffered saline (PBS), the aortas were carefully cut longitudinally and mounted *en face* (face down) on slides and then covered with cover slip for endothelial layer imaging. The images from DAF-2DA, DHE and DAPI staining were quantified with Image J software and results are presented as the ratio of DAF-2DA and DAPI positive nucleus or ratio of DHE and DAPI.

### Statistics

Data are given as mean ± SEM. In all experiments, n represents the number of experiments or animals. Statistical analysis was performed with Student's unpaired t-test or analysis of variance (ANOVA) with Bonferroni post-test. Differences in mean values were considered significant at two tailed P ≤ 0.05.
